# CRISPR/CAS9-Mediated Genome Editing of miRNA-155 Inhibits Proinflammatory Cytokine Production by RAW264.7 Cells

**DOI:** 10.1155/2015/326042

**Published:** 2015-11-30

**Authors:** Weixia Jing, Xuewu Zhang, Wenyan Sun, Xiujuan Hou, Zhongqiang Yao, Yuelan Zhu

**Affiliations:** ^1^Department of Medical Services, The Third Affiliated Hospital, Beijing University of Chinese Medicine, Beijing 100029, China; ^2^Department of Rheumatology and Immunology, Clinical Immunology Center, Peking University People's Hospital, Beijing 100044, China; ^3^Department of Pharmacology, School of Chinese Medicine, Beijing University of Chinese Medicine, Beijing 100102, China; ^4^Department of Rheumatology, Dongfang Hospital, Beijing University of Chinese Medicine, Beijing 100078, China; ^5^Department of Rheumatology & Immunology, Peking University Third Hospital, Beijing 100191, China

## Abstract

MicroRNA 155 (miR-155) is a key proinflammatory regulator in clinical and experimental rheumatoid arthritis (RA). Here we generated a miR-155 genome knockout (GKO) RAW264.7 macrophage cell line using the clustered regulatory interspaced short palindromic repeat (CRISPR)/CRISPR-associated protein 9 (CAS9) technology. While upregulating the Src homology-2 domain-containing inositol 5-phosphatase 1 (SHIP1), the miR-155 GKO line is severely impaired in producing proinflammatory cytokines but slightly increased in osteoclastogenesis upon treatment with receptor activator of nuclear factor-*κ*B ligand (RANKL). Taken together, our results suggest that genome editing of miR-155 holds the potential as a therapeutic strategy in RA.

## 1. Introduction

Rheumatoid arthritis (RA) reportedly affects over 21 million people worldwide [1]. RA is an autoimmune inflammatory disease affecting joints. It is characterized by macrophage and lymphocyte infiltration, proliferation of synovial fibroblasts, and ultimate joint destruction [2]. Macrophages play an important role in RA pathogenesis [3]. The number of macrophages is higher in the inflamed synovial membrane in RA than in normal joints and positively correlates with the severity of joint pain and inflammation [4, 5]. While a number of drugs have been approved in treating RA, gene or cell therapy has not been extensively explored.

MicroRNA 155 (miR-155) is found within the BIC gene [6] on Chromosome 16 in mouse and Chromosome 21 in human [7]. miR-155 has been linked to the pathogenesis of RA in clinical and experimental models as it is upregulated in synovial membrane and synovial fluid macrophages from patients with RA [8]. Knockdown (KD) of miR-155 by siRNA inhibited the production of proinflammatory cytokines [9]. The mechanism by which miR-155 participates in the formation of RA may be multifaceted, one of which is that miR-155 targets the 3′ untranslated region of Src homology-2 containing inositol phosphatase 1 (SHIP1), a negative regulator of inflammation [10]. Consequently, increased miR-155 in RA leads to reduced levels of SHIP1, resulting in heightened production of proinflammatory cytokines [8].

We sought to manipulate miR-155 expression by the CRISPR/CAS9 genome editing technology in order to alleviate inflammation in RA. The CRISPR system relies on CRISPR RNAs (crRNAs) in complex with CRISPR-associated (Cas) proteins to direct degradation of complementary sequence present within invading viral and plasmid DNA [11]. Recent studies have optimized and engineered the system so that the CAS9 protein can be directed by individual guide RNA (gRNA) to modify genomic target locus. In other words, this bacterial type II CRISPR system is now adapted for specific modification of genomic DNA in human cells [12–14]. This new class of genome engineering tool will help to create new cell lines for basic research and facilitate gene therapy with advances in primary cell and stem cell technology [15].

In this study, we successfully mutated the endogenous miR-155 gene in murine macrophage cell line RAW264.7 using CRISPR/CAS9 technology and obtained miR-155 genome knockout (GKO) clones. Further analyses showed that miR-155 GKO clones expressed higher levels of SHIP1 but produced much less proinflammatory cytokines upon LPS stimulation.

## 2. Materials and Methods

### 2.1. Cell Lines and Reagents

Murine macrophage cell line RAW264.7 (ATCC TIB-71) and human kidney epithelial cell line 293T (ATCC CRL-3216) were maintained in DMEM supplemented with 5% penicillin and streptomycin and 10% heat-inactivated fetal bovine serum (Gibcol). RANKL was purchased from Peprotech EC. Mouse TNF-*α*, IL-6, IL-12p70, and IL-12p40 ELISA Kits were purchased from Thermo Scientific.

### 2.2. DNA Constructs

The lentiviral packaging plasmid pCMV-ΔR8.2 (*Addgene plasmid 12263*) and pCAG-VSVG were obtained from Addgene (Plasmid #35616). The lentiviral vector pLentiCRISPR, which expresses CAS9 and gRNA, was obtained from Addgene as well. The gRNA that targets mouse miR-155 genomic sequence was subcloned into the lentiCRISPR vector by following the instruction. The gRNA sequence is 5′-TAGTGTTAATCGTAATTGTC-3′.

### 2.3. Verification of gRNA-Mediated Genome Cleavage

RAW264.7 cells were transfected with empty lentiCRIPSR or lentiCRISPR containing gRNA that targets miR-155. 72 hours after transfection, genomic DNA was isolated for PCR amplification of a 545 bp fragment that encompasses the mature miR-155 region using the following primers: F: 5′-ACTTAGAAGCATTTCAGAGCTC-3; R: 5′-GATACAAGTTTCACTTTCCATTC-3′. Fragments amplified from wild type cells and potentially mutated cells were purified and reannealed at 1 : 1 ratio followed by digestion with T7 endonuclease I (NEB). Digested products were separated on a 1% agarose gel for imaging.

### 2.4. Generation of miR-155 GKO Clones

1 × 10^6^ 293T cells were transfected with 1 *μ*g pLentiCRISPR containing miR-155 gRNA, 1 *μ*g pCMV-ΔR8.2, and 0.5 *μ*g pCAG-VSVG to package virus. 1 × 10^5^ RAW264.7 cells were then infected with 100 *μ*L lentivirus for two days. After limiting dilution, single clone was selected out in the presence of puromycin (2 *μ*g/mL) for three weeks. To determine the mutation, genomic DNA was purified and sequenced.

### 2.5. Quantitative Real-Time PCR

MicroRNAs were isolated using mirVana miRNA Isolation Kit (Life Technologies). Mature miR-155 was quantified with the TaqMan microRNA assay kit for mmu-miR-155 (Applied Biosystems, Foster City, CA) following the manufacturer's instruction.

### 2.6. Western Blotting

RAW264.7 cells were lysed with a lysis buffer (50 mM Tris-HCl, pH 7.5, 150 mM NaCl, 1% NP-40, 1 mM DTT) supplemented with a protease inhibitor mixture (Sigma) on ice. 20 *μ*g lysates were separated on a 9% SDS-polyacrylamide gel (SDS-PAGE) for western blotting. Transferred blots were then incubated with the rabbit anti-SHIP1 antibody (1/500) (sc-8425, Santa Cruz Biotechnology, Inc., Santa Cruz, CA) or the anti-*β*-actin antibody (Abcam, ab8227) overnight at 4°C. The secondary antibody used in the western blotting was a 1 : 2000 dilution of HRP-linked anti-IgG, followed by detection using the ECL reagents (Pierce).

### 2.7. Measurement of Osteoclastogenesis

A detailed protocol has been published elsewhere [16]. To measure TRAP activity, RANKL-treated RAW264.7 cells were first fixed in 4% (v/v) paraformaldehyde for 10 min, washed in 95% (v/v) ethanol, and then incubated in 100 *μ*L of phosphatase substrate (3.7 mM p-nitrophenyl phosphate, pH 4.6) at room temperature for 30 min. After the incubation, the reactions were stopped by adding 0.1 mL of 0.1 N NaOH, and the absorbance at 410 nm was determined with a SpectraMax (Molecular Devices) microplate reader. In addition to TRAP activity assay, cells were stained for TRAP with 0.1 mg/mL naphthol AS-MX phosphate and 0.6 mg/mL fast red violet LB salt in 0.1 M sodium acetate buffer (pH 5.0). TRAP-positive cells with three or more nuclei were counted. Images were collected using an inverted Olympus IX-81 microscope.

### 2.8. miR-155 Mimic and Inhibitor

miR-155 mimic (dsRNA oligonucleotides) and control mimic were purchased from GenePharma (Shanghai, China). RAW264.7 cells were transfected using lipofectamine RNAiMAX (Invitrogen) according to the instruction at a final concentration of 5, 10, or 25 nM.

### 2.9. Statistical Analysis

Student's *t*-tests were performed on all experiments and a *p* value <0.05 is considered statistically significant.

## 3. Results

### 3.1. Construction of gRNA That Targets Mouse miR-155

**Figure 1:**
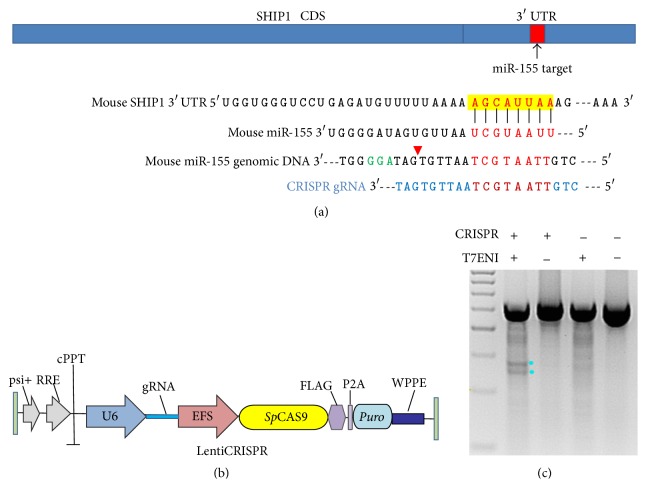
miR-155 genome knockout strategy. (a) Gene structure of mouse SHIP1 and miR-155 targeting sequence within 3′-UTR. The seed sequences of miR-155 were in red and the PAM sequence within miR-155 genomic DNA was in green. gRNA sequence was highlighted in blue. The predicted CAS9 cutting site was indicated with a red triangle. (b) Structural illustration of lentiCRISPR vector. (c) T7 endonuclease I assay for CRISPR efficiency. Bands representing indel formation were indicated with two light blue dots.

Mature miR-155 is highly conserved among species with a “seed sequence” containing 8 nucleotides. This “seed sequence” complements that within the 3′-untranslated region (UTR) of SHIP1 (Figure 1(a)). Ideally, one would mutate the miR-155 “seed sequence” to ablate its effect. Recognition of genomic DNA by streptococcus CAS9 protein requires a PAM sequence, which is immediately downstream of the sequences targeted by the gRNA [17]. The nearest PAM sequence within the mouse miR-155 genome is 9 nucleotides downstream of its “seed sequence” (Figure 1(a)). As CAS9 has been known to cut the DNA 3 nucleotides upstream of the PAM sequence, such a gRNA design is not expected to alter the “seed sequences,” but rather the random deletion or insertions as a result of nonhomologous end joint (NHEJ) after CAS9 cleavage may significantly shorten or alter the pre-miR-155 sequences so that the production of mature miRNA-155 may be reduced. The gRNA was subcloned into the recently published lentiCRISPR vector that drives the expression of both CAS9 and gRNA (Figure 1(b)) [12]. This vector allows the packaging of lentivirus for high efficiency delivery into cell lines. In order to examine whether the gRNA leads to the cleavage of genome DNA, we transfected the vector into RAW264.7 cell line and performed T7 endonuclease I assay. As shown in Figure 1(c), 72 hours after transfection, indels were observed, suggesting that our construct indeed led to cutting of genomic DNA.

### 3.2. miR-155 Genome Editing Upregulated SHIP1 in RAW264.7 Cells

**Figure 2:**
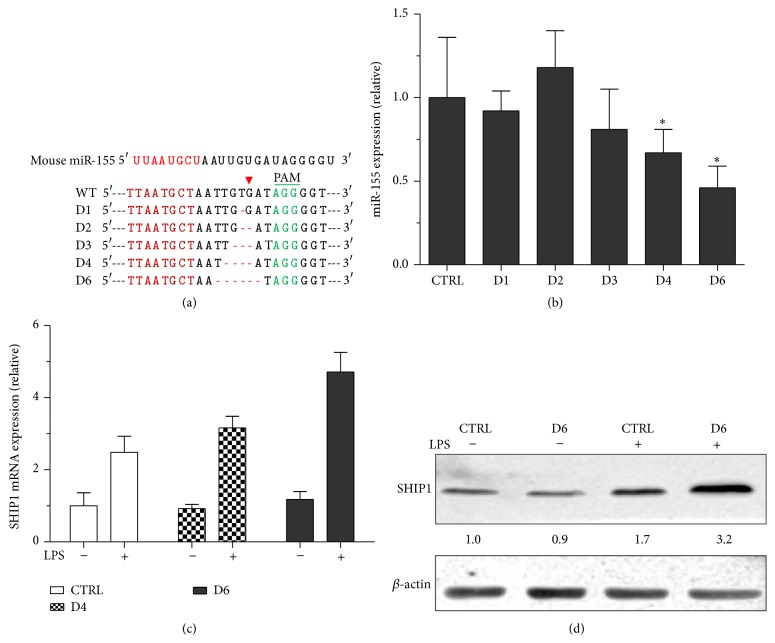
Generation of miR-155 GKO clones. DNA sequencing results of wild type and different deletions of miR-155 genomic DNA were presented in (a). (b) RT-qPCR results of mature miR-155 in different clones. ^*∗*^ *p* < 0.05. (c) SHIP mRNA was quantitated by RT-qPCR from LPS-treated RAW264.7 cells or D4 or D6 clones. (d) RAW264.7 cells were stimulated by LPS (100 ng/mL) for 36 hours followed by western blotting for SHIP1. The numbers below SHIP1 indicate the normalized expression levels.

Next, we packaged lentivirus to mutate endogenous miR-155 gene. RAW264.7 cells were infected and then selected in the presence of puromycin (2 *μ*g/mL). After three weeks, a number of clones were obtained and then sequenced. We categorized the derived mutants into five groups, with one to up to six nucleotide deletions near the predicted CAS9 cutting site (Figure 2(a)), namely, D1 to D6. Of note, we did not obtain insertional mutant in our study. We subsequently isolated RNA from the derived clones and determined the abundance of mature miR-155 using TaqMan RT-PCR. As indicated in Figure 2(b), deletion of one to three nucleotides failed to reduce the miR-155 levels. Deletion of four nucleotides weakly reduced and then D6 significantly reduced the production of mature miR-155 (Figure 2(b)).

SHIP1 is a known target by miR-155. SHIP1 negatively regulates the production of proinflammatory cytokines by macrophages. To assess the effect of miR-155 GKO on SHIP1 expression, we performed RT-qPCR and found that while LPS stimulation induced SHIP1 mRNA expression, D4 and D6 clones showed reduced expressions of SHIP1 mRNA (Figure 2(c)). In consistence, western blotting analysis confirmed that D6 clone produces more SHIP1 protein in response to LPS stimulation in comparison to the WT cells (Figure 2(d)).

### 3.3. Decrease in Proinflammatory Cytokine Production in miR-155 GKO Cells

**Figure 3:**
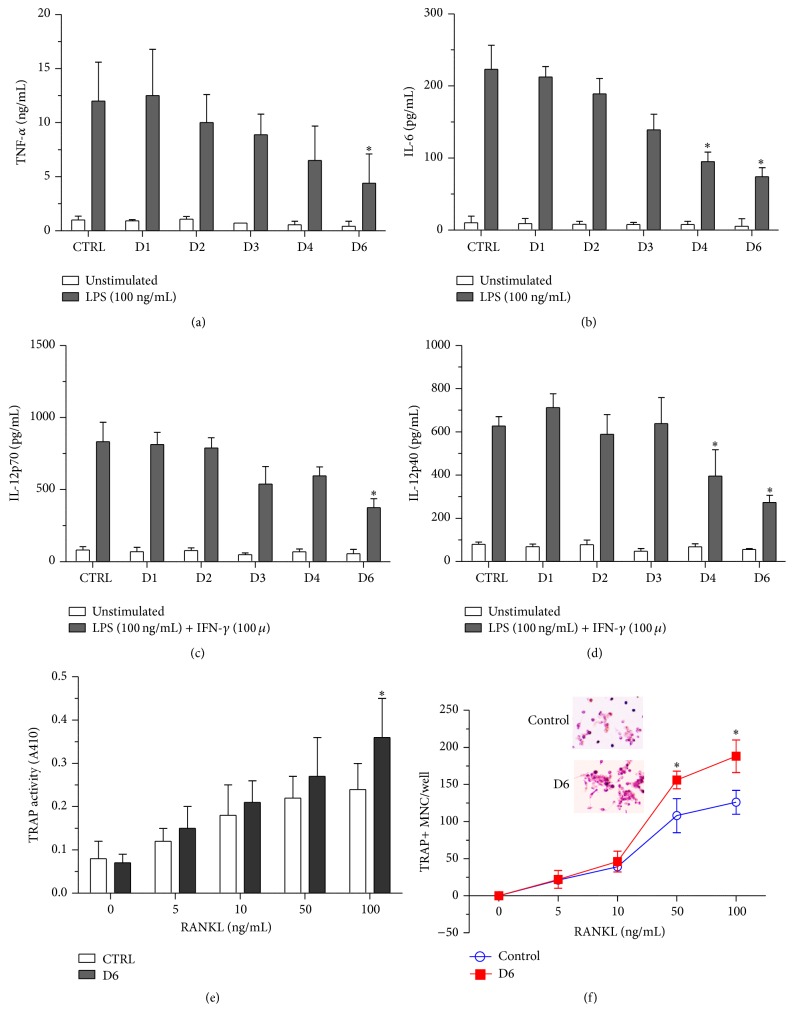
Cytokine production profiles of miR-155 GKO clones. RAW264.7 or indicated clones were stimulated with 100 ng/mL LPS ((a) and (b)) or LPS + IFN-*γ* (100 u) ((c) and (d)) for 48 hours. Supernatants were collected for ELISA analyses. ^*∗*^ *p* < 0.05. (e) cells were treated with RANKL at indicated concentrations. TRAP activity from each well was measured as described in Section 2.  ^*∗*^ *p* < 0.05. (f) TRAP-positive multinucleated cells (MNC) were counted and plotted. ^*∗*^ *p* < 0.05. Inset shows representative images of wild type RAW264.7 cells (top panel) or D6 clone (bottom panel) which have differentiated into TRAP-positive osteoclasts after RANKL stimulation (100 ng/mL).

One of the characteristics in RA patients is the chronic production of proinflammatory cytokines due to autoimmunity. To assess the ability of miR-155 GKO clones to produce cytokines, cells were stimulated by LPS or LPS + IFN-*γ*. In general, D1–D3 clones showed a trend of reduced production of TNF-*α*, IL-6, and IL-12, although the reduction was not statistically significant. By contrast, D4 clone and especially D6 clone produced much less cytokines upon cell stimulation (Figure 3). These results not only bolster the previous notion that miR-155 plays a proinflammatory role in macrophages but also support our original hypothesis that modifying genomic miR-155 sequence may be of therapeutic value in alleviating inflammation in RA.

### 3.4. Increase in Osteoclastogenesis in miR-155 GKO Cells

One of the hallmark characteristics in RA patients is the increased osteoclastic activity. RAW264.7 has been used as the model system to study osteoclastogenesis because RANKL treatment is known to increase the TRAP enzymatic activity, the key marker of osteoclast differentiation, and subsequently induce differentiation and the formation of multinucleated osteoclasts [18]. To test the effect of miR-155 GKO on osteoclastogenesis, we treated WT RAW264.7 or D6 clone with RANKL at different concentrations. As shown in Figure 3(e), increasing doses of RANKL elevated TRAP activity in WT RAW264.7 cells. The induction trend was further enhanced in D6 clone. We then directly enumerated the formation of multinucleated TRAP-positive cells from both groups. The numbers of multinucleated cells in RANKL-treated D6 clone also slightly increased (Figure 3(f)).

### 3.5. miR-155 GKO Can Be Rescued by Introduction of miR-155 Mimic

**Figure 4:**
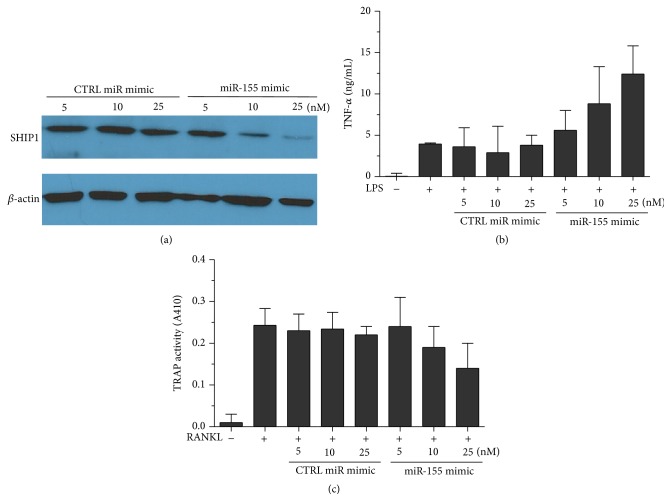
miR-155 mimic reintroduced miR-155 effect in GKO cells. (a) D6 clone was transfected with CTRL or miR-155 mimics at indicated concentrations and then further stimulated with LPS (100 ng/mL) for 36 hours. SHIP1 protein was quantified by western blotting. (b) and (c) D6 clone was transfected with indicated mimics and stimulated with LPS (100 ng/mL) for 48 hours. TNF-*α* production and TRAP activities were determined.

One caveat with the CRISPR/CAS9-mediated genome editing is its off-target effect [19]. To ensure that the observed decrease in mature miR-155 in this study was due to GKO, we transfected the D6 clone with miR-155 mimic that resembles the mature miR-155. The miR-155 mimic is RNA and hence not subjected to the continuous cleavage by CAS9. The small oligo also displayed exceedingly high transfection efficiency. As illustrated in Figure 4(a), transfection of D6 clone with miR-155 mimic, but not a control mimic, successfully repressed the SHIP1 protein synthesis. Furthermore, miR-155 mimic increased the production of TNF-*α* in D6 clone (Figure 4(b)) and decreased the TRAP activity (Figure 4(c)). Altogether, miR-155 mimic is able to reintroduce the effect of miR-155 into D6 clone, suggesting that our findings are due to the direct knockout of miR-155.

## 4. Discussion

A comprehensive list of experimentally validated miR-155 targets has been published [20]. Relevant to RA, miR-155 has been reported to regulate a number of genes, including PU.1 [21, 22], AID [23], SHIP1 [10], SMAD5 [24], and SOCS1 [25], all of which are known regulators of inflammation. Of note, the role of miR-155 in mediating inflammatory response has been multifaceted. Bala et al. reported that upregulation of miR-155 in macrophages contributes to increased TNF-*α* [9], whereas Wang et al. published that inducible miR-155 was responsible for the induction of type I IFNs by targeting SOCS1 [25]. The multifunctional role of miR-155 is not completely unexpected given the number of targeted genes that miR-155 regulates. Here, by using miR-155 GKO clones, we provided direct evidence that removal of miR-155 results in decrease in proinflammatory cytokine production by macrophages, therefore confirming the previous observation that elevated miR-155 contributes to the sustained levels of cytokine production in RA patients. Due to limited resources, we were unable to perform deep sequencing analysis on the miR-155 GKO clone for identification of all off-targets. Nevertheless, we were able to reintroduce the miR-155 effect by transfecting miR-155 mimic back to the GKO clone. Altogether, these results suggest that the mutated endogenous miR-155 gene perhaps led to truncated pre-miR-155 products, which failed to mature into shorter but stable miR-155.

Previously a group has demonstrated that lentivirus-mediated silencing of miR-223 ameliorated collagen-induced arthritis in mice [26], raising great interest in developing gene therapy to treat RA. Here we demonstrated that mutating genomic miR-155 is another way of relieving the inflammation. The potential problem associated with this strategy, however, is that miR-155 GKO slightly enhanced the production of TRAP activity and promoted the differentiation into osteoclasts. Previously, miR-155 was found to inhibit RANKL-induced osteoclastogenesis by repressing the expression of MITF in RAW264.7 cells [27]. Interferon-*β* also reportedly induced miR-155 expression which then inhibits osteoclast differentiation by targeting SOCS1 and MITF [28]. Given that osteoclastogenesis is an important event during RA pathogenesis, perhaps miR-155 GKO can be employed early in the disease process as a means to alleviate the production of proinflammatory cytokines.

Lentivirus transduction is highly efficient and offers long-term effect. In case transient delivery of CRISP/CAS9 is desired, adeno-associated virus (AAV) may be deployed as it has recently been demonstrated to deliver CAS9 and gRNA into primary cells [29, 30]. AAV vectors are even safer in gene therapy because they do not integrate into the host genome. Another limit of our study is that RAW264.7 is a cell line, not the primary synovial macrophages. Moreover, many cell types are implicated in RA pathogenesis [31]. While it is unclear how the lentivirus-mediated miR-155 knockout will affect T cell response and autoantibody production, our proof-of-principle study demonstrated the potential of developing therapeutic strategy by nullifying miR-155 to alleviate RA. Further investigation of the therapeutic efficacy of miR-155 GKO* in vivo* is under way.
